# Recent Advances in Serum Biomarkers for Risk Stratification and Patient Management in Cardio-Oncology

**DOI:** 10.1007/s11886-022-01834-x

**Published:** 2023-02-15

**Authors:** Pouya Joolharzadeh, Mario Rodriguez, Raja Zaghlol, Lauren N. Pedersen, Jesus Jimenez, Carmen Bergom, Joshua D. Mitchell

**Affiliations:** 1 General Medical Sciences, Washington University School of Medicine, St. Louis, MO USA; 2 Cardiovascular Division, Department of Medicine, Washington University School of Medicine, St. Louis, MO USA; 3 Cardio-Oncology Center of Excellence, Washington University School of Medicine, St. Louis, MO USA; 4 Department of Radiation Oncology, Washington University School of Medicine, St. Louis, MO USA; 5 Alvin J. Siteman Cancer Center, Washington University in St. Louis, St. Louis, MO USA

**Keywords:** Cardiotoxicity, Cancer therapy, Cardio-oncology, Biomarkers, Screening, Risk prediction

## Abstract

**Purpose of Review:**

Following significant advancements in cancer therapeutics and survival, the risk of cancer therapy-related cardiotoxicity (CTRC) is increasingly recognized. With ongoing efforts to reduce cardiovascular morbidity and mortality in cancer patients and survivors, cardiac biomarkers have been studied for both risk stratification and monitoring during and after therapy to detect subclinical disease. This article will review the utility for biomarker use throughout the cancer care continuum.

**Recent Findings:**

A recent meta-analysis shows utility for troponin in monitoring patients at risk for CTRC during cancer therapy. The role for natriuretic peptides is less clear but may be useful in patients receiving proteasome inhibitors. Early studies explore use of myeloperoxidase, growth differentiation factor 15, galectin 3, micro-RNA, and others as novel biomarkers in CTRC.

**Summary:**

Biomarkers have potential to identify subclinical CTRC and may reveal opportunities for early intervention. Further research is needed to elucidate optimal biomarkers and surveillance strategies.

## Introduction

Over 18 million patients worldwide were diagnosed with cancer in 2020 [1]. With significant advancements in cancer therapeutics over the last few decades, the prognosis for these patients has improved dramatically. Yet, as patients with cancer live longer, it is increasingly recognized that they have a considerable cardiovascular disease burden with a 2- to 6- fold higher cardiovascular mortality rate than the general population [2]. Cardiovascular disease and cancer often coexist due to their high prevalence, shared risk factors (e.g., smoking), bidirectional relationship (e.g., inflammation), and the potential cardiotoxic effects of cancer treatments. Not only does cardiovascular disease affect the quality of life and survival of patients after cancer treatment, but cardiac events during therapy can lead to discontinuations of treatment and worsen cancer survival.

Since cardiovascular complications are a significant contributor to morbidity and mortality in cancer patients and survivors, several cardiac biomarkers have been evaluated for baseline risk stratification, early recognition of subclinical disease during and after cancer therapy, and prognostication. The ideal cardiac biomarker should be easily measured, reproducible, and reliably predict clinical outcomes. It should also influence management plans by directing either preventive (risk stratification) or treatment (disease detection) regimens. In a seminal paper, Cardinale et al. showed not only that elevated troponin I measured soon after high-dose chemotherapy could detect patients at risk for further left ventricular (LV) dysfunction, but also initiation of enalparil after troponin elevation could prevent LV decline [3]. The efficacy of biomarkers for any given cancer therapy regimen will vary with the mechanism of toxicity and biomarker pathophysiology, though biomarkers can also have more universal applications such as the use of troponin and natriuretic peptides to detect myocardial necrosis/injury and chamber dilation/volume overload, respectively.

This article will review recommendations for biomarker use throughout the cancer care continuum, the underlying pathophysiology of major biomakers, and the current state of the evidence for the utility of biomarkers in risk stratification and disease detection across different cancer types and cancer treatmements. Though cancer therapy-related cardiotoxicity (CTRC) includes a broad range of conditions such as radiation induced valvular disease or atherosclerosis, arrhythmias, and pulmonary hypertension, biomarkers are most commonly used for detection of LV dysfunction and cardiomyopathy. The most investigated and validated biomarkers are troponin and natriuretic peptides (NP), with several emerging biomarkers under study.

## Biomarker Utilization at Time of Cancer Diagnosis

At the time of initial diagnosis, baseline biomarker measurement (troponin and NP) can complement a patient’s cardiovascular history and physical examination to help detect those at increased risk of developing cardiac dysfunction or those with pre-existing subclinical disease. These patients may benefit from cardioprotective medications and/or more frequent monitoring during or following cancer treatment. Biomarkers measured at baseline also provide a helpful reference for patients with possible cardiotoxicity after initiating cancer treatment. While acknowledging that further study is needed, the European Society of Medical Oncology (ESMO) recommends considering assessment of baseline biomarkers (troponin, NP) in higher-risk patients (e.g., relapsed myeloma or planned anthracycline-based chemotherapy) to help identify patients at increased risk for LV dysfunction or heart failure (HF) [4]. The European Society of Cardiology (ESC) and International Cardio-Oncology Society (ICOS) have also published a collaborative expert consensus statement unanimously supporting baseline biomarker measurement (troponin, NP) to aid in risk assessment [5]. More recently, the ESC, European Hematology Association, the European Society for Therapeutic Radiology and Oncology, and ICOS published collaborative cardio-oncology guidelines recommending baseline biomarker assessment (troponin, NP) [6••]. Comparisons between consensus recommendations and guidelines are detailed in Table 1.

**Table 1:** Biomarker recommendations in cardio-oncology by cancer therapy and organization

**Organization**	ESMO [4]	HFA (ESC) and ICOS [5]	ESC [6••]
Anthracyclines	**High risk (pre-existing significant cardiovascular disease):** Consider NP/cTn at baseline (Level III, Grade A)	**Low risk:** NP/cTn at baseline and 12 M post-treatment. Optional before 5th cycle during treatment	**Low/moderate risk:** NP/cTn at baseline, C2/C4/C6, and 3 M post-treatment (Class IIa, Level C)
	**Asymptomatic surveillance with normal EF:** NP/cTN **e**very 3–6 weeks (Level III, Grade C)	**Medium risk:** NP/cTN at baseline, before C5, and 12 M post-treatment. Optional before every cycle	**High/very high risk:** NP/cTn at baseline, C1–C6, and then 3 M and 12 M post-treatment (Class I, Level B)
	**Asymptomatic surveillance with LVEF decrease ≥ 10% from baseline to 50% or decrease in LVEF ≥ 40% but < 50%:** consider NP/cTn after each dose (Level III, Grade A)	**High risk:** NP/cTN at baseline, before C2, C4, and C6. 3-6 M and 12 M post-treatment. Optional before every cycle	
Anti-HER2 therapy	**Surveillance in adjuvant therapy**: NP/cTn can be considered during surveillance — no specified timing (Level III, Grade C)	**Early invasive HER2 + breast cancer with neoadjuvant or adjuvant trastuzumab** **Low risk:** NP/cTn at baseline and every 4 cycles, optional 6–12 months post-treatment **Medium risk:** NP/cTn at baseline, before alternate cycles for 3–6 M and then every 3 cycles in the first year. 3–6 M post-treatment and optional 12 M post-treatment **High risk:** NP/cTn at baseline, before alternate cycles for 3–6 M then every 3 cycles in the first year, and 3 and 12 M post-treatment	**Low/moderate risk:** NP/cTN at baseline (Class IIa, Level A), every 3 M during treatment, and then 12 M post-treatment (Class IIb, Level C)
	**Asymptomatic surveillance in metastatic disease:** periodic cardiac evaluation, potentially including cardiac biomarkers (Level I, Grade B)		**High/very high risk:** NP/ cTN at baseline (Class I, Level C), every 2–3 cycles during therapy, and then 3 M and 12 M post-treatment (Class IIa, Level C)
	**Asymptomatic surveillance with LVEF decrease 10% from baseline or drop to ≥ 40% but < 50%:** consider NP/cTn monthly (Level III, Grade A)		
	**HER2 therapy interruption**: if LVEF ≥ 40% and no HF symptoms, resumption of HER2 therapy should be considered and supported by cardiac biomarker assessment (Level III, Grade B)		
		**Metastatic HER2 + breast or gastric cancer with long-term HER2 therapy** **Low risk:** NP/cTn at baseline. Post-treatment only if symptomatic **Medium risk:** NP/cTn at baseline and every 4 months. Post-treatment only if symptomatic **High risk:** NP/cTn at baseline, before every cycle for 3–6 M then every 3 cycles in the first year. Post-treatment only if symptomatic	
VEGF inhibitors	**Asymptomatic surveillance:** Serial monitoring is recommended which can include cardiac biomarkers and/or imaging (Level I, Grade A)	**Low risk:** NP at baseline and every 3 months during treatment	**Moderate risk:** NP at baseline and every 4 months during the first year (Class IIb, Level C)
	**Symptomatic:** cardiac biomarkers should be considered (Level III, Grade A)	**Medium/high risk:** NP at baseline, 2–4 weeks after starting treatment, and every 3 months during treatment	**High/very high risk:** NP at baseline, 4 weeks after starting therapy, then every 3 M in the first year (Class IIa, Level C)
Immune checkpoint inhibitors	**Symptomatic, incidental arrhythmia, or LV systolic dysfunction:** NP/cTn (Level IV, Grade C)	**Low risk:** NP/cTn at baseline	NP/cTn at baseline in all patients (I, B). Serial cTn considered before doses 2, 3, and 4 and if normal, reduced to every 3 doses until therapy completion (Class IIa, Level B)
		**High risk:** NP/cTn at baseline, before C2–C4. If C4 is normal, measure at alternate doses between C6 and C12. If still normal, reduce to every 3 months	
Proteasome inhibitors	-	NP at baseline and for the first few cycles (particularly with carfilzomib)	**Low/moderate risk:** NP considered at baseline (Class IIa, Level C)
			**High/very high risk:** NP at baseline (Class I, Level C)
			**Carfilzomib/bortezomib:** NP at baseline and during C1–C6 (Class IIa, Level B)
Cardiac amyloid	-	-	NP/cTn at baseline for the diagnosis of AL-CA in patients with plasma cell dyscrasia (Class I, Level B). NP/cTn at baseline and every 3–6 M for AL-CA on proteasome inhibitors (Class I, Level B)
Carcinoid heart	-	-	NP screening considered every 6 M (Class IIa, Level B)
Radiation	-	Discussed without specific recommendations	No biomarker recommendations or discussion

Recommendations by society for patients on potentially cardiotoxic cancer therapies. ESMO recommendations are presented as (Level of evidence, Grade of recommendation). Levels of evidence represented are I (at least one large RCT), III (prospective cohort study), and IV (retrospective cohort study). Grades of recommendation represented are A (strongly recommended), B (generally recommended), and C (optional). ESC recommendations are presented as (Class of recommendation, Level of evidence). Classes of recommendation are Class I (indicated), Class IIa–b (considered), and Class III (not recommended). Levels of evidence are Level A (multiple randomized trials or meta-analysis) to Level C (consensus opinion or small retrospective studies). For risk level definitions, see Table 2

*C* cycle (i.e., C2 is cycle 2), *cTn* cardiac troponin, *ESC* European Society of Cardiology, *ESMO* European Society of Medical Oncology, *HER* human epidermal receptor, *HFA* Heart Failure Association of the ESC, *ICOS* International Cardio-Oncology Society, *NP* natriuretic peptide, *VEGF* vascular endothelial growth factor

The best supporting evidence for baseline biomarker assessment for risk assessment or prognostication comes from studies in patients with relapsed myeloma, those receiving anthracycline or anti-human epidermal growth factor receptor 2 (HER2) therapy, and those with amyloidosis. In patients with relapsed myeloma starting carfilzomib therapy, baseline elevation with brain natriuretic peptide (BNP) or N-terminal proBNP (NT-ProBNP) conferred an 11-fold risk for cardiovascular adverse events on treatment [7]. In a study of 452 patients from the Herceptin Adjuvant (HERA) study, baseline troponin elevation conferred a 4.5-fold increased risk for significant LVEF drop following trastuzumab therapy [8]. In patients with AL amyloidosis, biomarker assessment with troponin and NPs are integral to disease staging and survival prognostication as a way to assess for the degree of cardiac involvement [9].

There is no evidence that elevated baseline biomarkers in isolation should influence the decision for, or choice of, cancer treatment. Conversely, baseline risk assessment using biomarkers can be valuable to guide monitoring and screening intervals, cardiovascular risk factor optimization, and potentially identify those most likely to benefit from cardioprotective medications as an adjunct to their cancer treatment. Cancer therapy decisions should always be done in a multidisciplinary fashion (including Hematology/Oncology and Cardiology) with appropriate recognition of the potential survival prolonging benefit of cancer therapy relative to the potential for, or even presence of, cardiovascular toxicity [10].

## Biomarker Utilization During Cancer Therapy

During cancer therapy, biomarkers have the potential to identify early subclinical cardiotoxicity before progression to overt cardiac dysfunction. In the aforementioned study by Cardinale and colleagues, elevation of troponin after high-risk chemotherapy identified patients at risk for future LV dysfunction [3]. More importantly, the use of enalapril mitigated subsequent LV dysfunction in patients with elevated troponin. ESMO recommends consideration for routine screening with troponin and NPs every 3–6 weeks during anthracycline treatment [4].

Biomarker assessment is generally recommended for consideration during anthracycline, anti-HER2 therapy, anti-vascular endothelial growth factor (VEGF) therapy, and other potentially cardiotoxic therapies, although data on the optimal screening protocols and patient selection are lacking [4]. There is increased interest for biomarker (troponin, NP) screening during treatment with immune checkpoint inhibitor (ICI) therapy for early detection of myocarditis, an uncommon, but potentially fatal complication of immunotherapy [5].

As in the general population, there is also clinical utility for the use of biomarkers (NP, troponin) as a diagnostic tool in a patient presenting with shortness of breath or other cardiac symptoms during or after cancer treatment. Separately, in patients noted to have a decline in LV function on screening echocardiogram, biomarkers can complement the cardiovascular examination to better quantify the clinical significance of the LV dysfunction [5]. Asymptomatic patients with normal biomarkers are much more likely to tolerate the decline in LV function compared to patients with symptomatic HF or marked increase in NP.

## Biomarkers During Cancer Survivorship

Biomarker utilization in cancer survivorship, particularly further out from cancer therapy, has been mainly investigated in adult survivors of childhood cancer. In 535 adults from the St. Jude cohort, 10 or more years out from cancer therapy and without history of cardiomyopathy, NT-ProBNP levels above the age- and sex-specific 97.5th percentile identified patients with a twofold increased risk for subsequent cardiomyopathy [11]. Only five survivors had elevated troponin levels. Theoretically, troponin, a marker of acute cardiac injury, would be less helpful during survivorship, while NPs could identify patients at risk for developing HF, similar to prior data showing utility in identifying subclinical HF in the general population [12]. Based on the significantly increased observed HF incidence, lifelong screening with echocardiogram is recommended by the Children’s Oncology Group for survivors of high-risk cancer therapy (e.g., anthracyclines, chest radiation) with reasonable cost-effectiveness [13]. Biomarkers have not been included in these survivorship recommendations to date. The ESMO guidelines recommend consideration for a CV evaluation that can include biomarkers following cardiotoxic cancer therapy at 6–12 months after therapy, at 2 years, and possibly periodically thereafter [4].

## Pathophysiology of Biomarkers

**Fig. 1:**
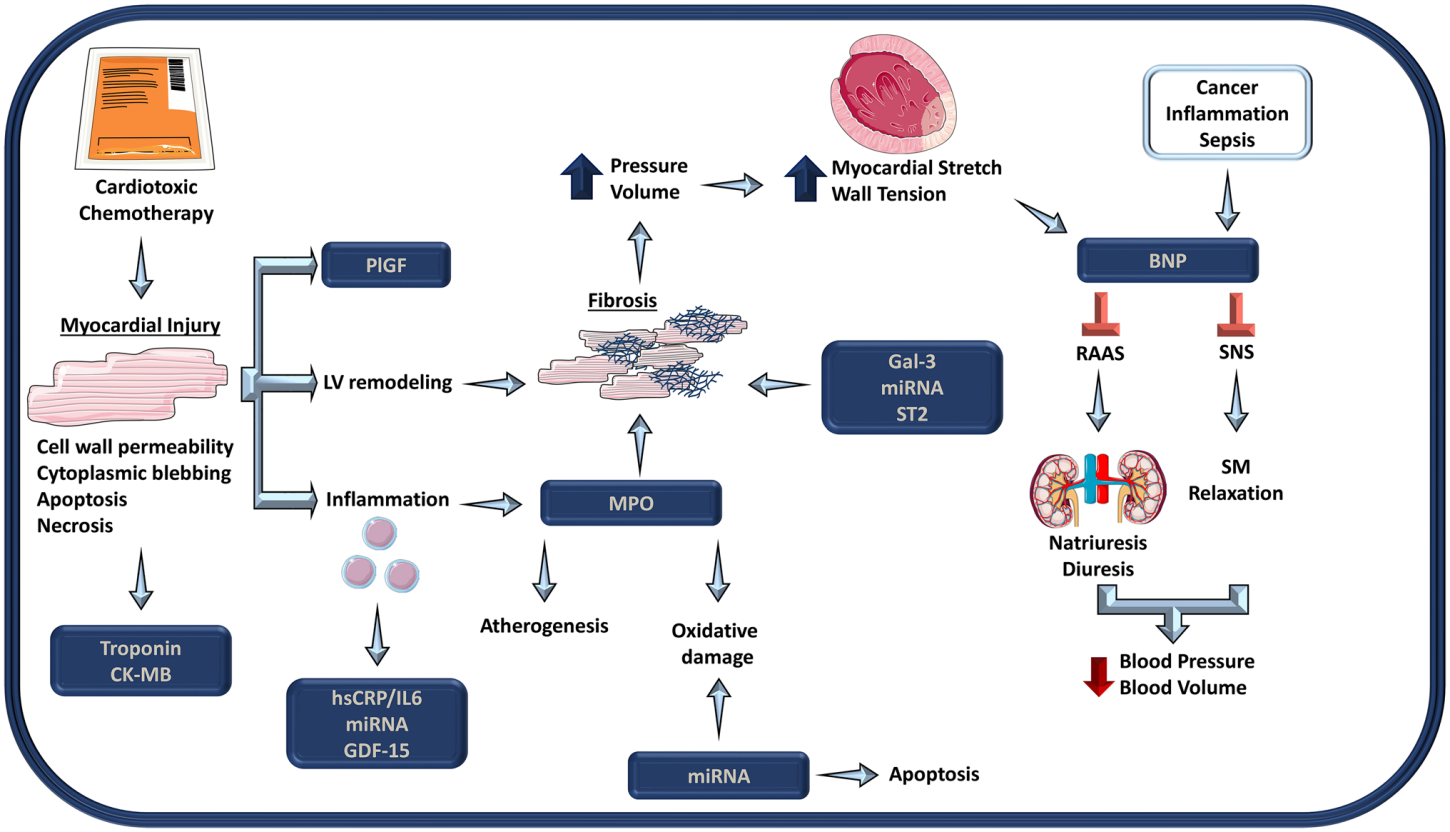
Pathophysiologic mechanisms of biomarkers implicated in cancer therapy related cardiotoxicity. These markers are reflective of myocardial injury (troponin, CK-MB, GDF-15), stretch and stress (BNP), inflammation (hsCRP, IL-6, GDF-15), oxidative stress (MPO, miRNA), fibrosis (MPO, gal-3, miRNA, ST2), and angiogenesis (PlGF). Of these markers, troponin and BNP are most commonly used in cardiac assessment. Whereas troponin is reflective of myocardial damage, BNP is reflective of stress culminating in neurohormonal changes that inhibit the renin-angiotensin system (RAAS) and sympathetic nervous system (SNS). This figure was partly generated using Servier Medical Art, provided by Servier, licensed under a Creative Commons Attribution 3.0 unported license (https://creativecommons.org/licenses/by/3.0/)

In this section, we briefly review the pathophysiology of established and emerging biomarkers and the mechanisms as they relate to general cardiovascular disease. Specific applications in cardio-oncology are discussed in subsequent sections (Fig. 1).

### Troponin

Markers of myocardial injury (troponin) were developed to diagnose acute coronary syndromes but have since been shown to have significant value in detecting cardiotoxicity from cancer therapy as well. Troponin acts as the regulatory complex of the cardiac myofibrillar apparatus and is critical in excitation-coupling [14]. While the troponin subunits are generally specific to the cardiac myocyte, emerging literature suggests that troponin T may be present in skeletal muscle as well, and thus, troponin I may be the most precise for cardiac damage [15].

Notably, elevations in troponin are not specific to an underlying clinical mechanism and do not necessarily reflect myocardial necrosis. In response to myocardial stress, troponin can be systemically detected due to transient increases in cell permeability from cellular wounds and cytoplasmic blebbing in addition to apoptosis (Fig. 1) [16]. More recently, the adoption of high-sensitivity cardiac troponin (hs-cTn) allows for the detection of very low troponin concentrations [17]. Thus, clinical scenarios like hypoxemia, anemia, tachyarrhythmias, shock, HF, and kidney disease can cause detectable hs-cTn levels.

### Natriuretic Peptides

Distinct to markers of myocardial injury, NPs are neurohormonal modulators that process sodium and water and usually increase with hemodynamic stress and congestion. Of the various NPs, brain natriuretic peptide (BNP) is the most clinically relevant in the diagnosis, treatment, and prognosis of HF as it predicts adverse outcomes at high concentrations [18, 19]. The predominant signal for BNP release is cardiac myocyte stretch in response to elevated intracardiac volume and pressure. It is also transcriptionally upregulated by catecholamines and angiotensin-II [20–23]. BNP is the biologically active peptide, while the remaining cleaved portion, NT-proBNP, is the inactive segment with a longer half-life [24]. When in its active form, BNP directly inhibits renin–angiotensin–aldosterone system (RAAS) and sympathetic nervous system (SNS) signaling, relaxes vascular smooth muscle cells, and relaxes mesangial cells, preventing renal tubular resorption of sodium. These mechanisms trigger vasodilation, natriuresis, diuresis, a reduction of circulating blood volume, and lower blood pressure (Fig. 1) [23, 25].

While BNP and NT-proBNP are most strongly related to cardiac myocyte stress, there are also emerging data showing a positive correlation between NT-proBNP and inflammatory cytokines like interleukin-6 (IL-6) [26]. In patients with cancer, BNP can be elevated in absence of clinical HF and positively correlates with high-sensitivity C-reactive protein (hsCRP), suggesting a relationship with cancer-related inflammation [27]. BNP can also be markedly elevated in severe sepsis and septic shock [28, 29]. The relationship between BNP, inflammation, and sepsis requires further investigation.

## Emerging Biomarkers

### Myeloperoxidase

MPO is an inflammatory enzyme produced and secreted by leukocytes in response to myocardial infarction. It generates reactive oxygen species and proteolytic enzymes that promote oxidative damage and extracellular matrix breakdown [30, 31]. This promotes atherogenesis and fibrosis, portraying risk for coronary artery disease (CAD) and HF [32, 33].

### Interleukin-6 and High-Sensitivity CRP

IL-6 is a cytokine that not only promotes hepatic production of CRP, but also activates endothelial cells, lymphocyte proliferation and differentiation, coagulation, and the hypothalamic–pituitary–adrenal axis. Both IL-6 and hsCRP are important inflammatory mediators that are cornerstones in the development of coronary artery disease [34]. HsCRP is well established to be predictive of coronary artery disease, MI, stroke, peripheral artery disease, and plaque instability in both primary and secondary prevention studies [35]. Similarly, IL-6 is independently associated with cardiovascular death, MI, all-cause mortality, and risk of hospitalization for HF within a cohort of over 14,000 patients in the STABILITY trial [36].

### Interleukin-1 Receptor-Like 1 (ST2)

Also known as Interleukin-1 receptor-like 1 (IL1RL1), ST2 is the receptor of the interleukin-1 (IL-1) family for the ligand IL-33 and is found in two forms, transmembrane and soluble ST2 [37]. Weinberg et al. first showed that ST2 is induced by myocardial stretch and myocardial injury [38]. Where transmembrane ST2 is protective against fibrosis, soluble ST2 sequesters IL-33 and negates these protective effects [37]. Higher levels of soluble ST2 have been shown to predict mortality in both acute and chronic HF [39, 40]. Though soluble ST2 is a relatively weak marker of acute myocardial injury, it is associated with development of HF in non-ST elevation ACS [41].

### Galectin-3

Galectin-3 is a member of the lectin family that is released during monocyte differentiation into macrophages and involved in multiple inflammatory signaling mechanisms [42]. It promotes fibroblast proliferation and is pro-fibrotic, inducing cardiac hypertrophy in rat models [43]. Gal-3 is also upregulated in many types of cancer, complicating its utility for detecting cancer therapy-related cardiotoxicity [42]. Gal-3 has shown promising, yet conflicting results as a prognostic marker for all-cause and cardiovascular mortality according to two meta-analyses [44, 45]. An analysis of the Penn Heart Failure Study found that gal-3 significantly correlated with severe HF symptoms and adverse events (all-cause mortality, and risk of cardiac transplantation or VAD placement) in a prospective cohort of chronic HF patients with preserved, reduced, and recovered ejection fraction. Interestingly, gal-3 alone may be a superior marker of adverse events in HF with preserved ejection fraction when compared to those with reduced ejection fraction. This effect is more pronounced when gal-3 is combined with NT-proBNP [44, 46].

### Placental Growth Factor

Placental growth factor (PlGF) is a member of the vascular endothelial growth factor family involved in hypertrophy and angiogenesis [47, 48]. It has been shown to be prognostic for all-cause mortality and non-fatal myocardial infarction in acute and long-term follow-up following acute coronary syndromes [49].

### Growth Differentiation Factor 15

Growth differentiation factor 15 (GDF-15) is a cytokine of the transforming growth factor-B family. It is released in response to sympathetic activation, ischemia, injury, cardiac remodeling, and inflammation and acts as a protective mechanism against norepinephrine-induced myocardial hypertrophy [50]. Clinically, GDF-15 has been shown to be prognostic for mortality in HF [51].

### Micro-RNA

Micro-RNAs (miRNA) are small and ubiquitous, non-coding RNAs that have important regulatory abilities. In the pathogenesis of HF, miRNAs have been involved in myocardial inflammation, apoptosis, hypertrophy, and fibrosis [52]. They have also been reported in acute myocardial infarction, arrhythmias, and pulmonary hypertension [53]. While yielding promise, miRNAs have not yet been found to be reliable biomarkers [54].

## Biomarker Assays

When interpreting and comparing biomarker research, it is important to recognize that there is significant variance in biomarker assays and normal reference ranges across different studies and cancer centers. Confounding this issue, traditional troponin assays have more recently been replaced by high-sensitivity assays, allowing for detection of only subtle troponin levels not previously possible in early cardiotoxicity studies. These issues impact generalizability of study findings and must be considered when translating research findings to clinical practice.

## Anthracycline-Induced Cardiotoxicity

Anthracyclines such as doxorubicin, daunorubicin, epirubicin, and idarubicin are often the treatment of choice for leukemia, lymphoma, sarcoma, and high-risk breast cancer. As topoisomerase II inhibitors, their cardiotoxic mechanisms include DNA damage, generation of reactive oxygen species, and iron-induced mitochondrial toxicity [55]. The risk of anthracycline-induced HF increases as the cumulative dose administered increases: 3–5% with 400 mg/m^2^ and as high as 18–48% at 700 mg/m^2^ [56]. A meta-analysis of patients treated with various doses of anthracyclines across 18 studies reported 6% developed clinical cardiotoxicity and 18% had subclinical cardiotoxicity over a median follow-up of 9 years [57]. Most cardiotoxicity develops in the first year after treatment, although childhood survivors have been noted to be at increased risk for cardiomyopathy throughout their lifetime [58].

### Troponin

There is a clear association between anthracycline-associated rise in troponin and future development of LV dysfunction. A 2020 meta-analysis by Michel et al. investigated the utility of troponins and/or NPs across 61 trials with 5691 patients [59••]. Anthracyclines and “high-dose chemotherapy” that included anthracyclines conferred the highest risk for troponin elevation (17.5-fold and 230.4-fold increased risk, respectively) [59••]. Patients with a troponin elevation after anthracycline treatment were subsequently at a sevenfold increased risk for LV dysfunction, with a 54% sensitivity, 79% specificity, and 93% negative predictive value [59••].

Across nine studies of various cancer treatments, preventive therapy with both ACEi/ARB and beta-blockers were associated with reduced troponin elevation on treatment [59••]. ACEi/ARB conferred a numerically stronger protective effect than beta-blockers. Interestingly, metoprolol has generally failed to show a protective benefit during anthracycline treatment, while carvedilol has shown benefit, potentially due to its antioxidant properties [60].

### Natriuretic Peptides

Limited by fewer studies, the same 2020 meta-analysis did not find conclusive evidence that elevations in NP levels with anthracycline (or other chemotherapy) could predict LV dysfunction [59••]. Across 4 anthracycline studies, NP levels were higher in patients with LV dysfunction vs those with preserved LVEF (standard mean difference 1.08) [59••]. Only one study of 52 patients evaluated whether an NP cutoff was associated with LV dysfunction, and no statistical difference was found [59••]. By its nature, troponin is a more specific marker of true myocardial injury, which can subsequently lead to future LV dysfunction, while rises in NP can also be associated with therapy associated fluid administration or other inflammatory mechanisms previously discussed. The value of NP may be in its ability to rule out cardiotoxicity, with a BNP < 100 ng/L having a negative predictive value of 92% for anthracycline-induced cardiotoxicity in the PREDICT study of 586 patients [61].

### Recommendations

The 2022 ESC guidelines recommend measuring NP and troponin in those at high and very high risk of CTRC at baseline, before every cycle during treatment, and 3 and 12 months following therapy completion. Those at low risk can be monitored at baseline, potentially every two cycles during treatment, and potentially at 3 months following therapy completion (Tables 1 and 2) [6••, 62•].

**Table 2:** HFA-ICOS and ESC risk assessment in patients receiving potentially cardiotoxic cancer therapy

**Baseline HFA-ICOS risk assessment tool** [62•] **(Tool specific to cancer therapy, examples listed for anthracyclines)**	**Risk following treatment**
**Very high risk**: Baseline HF or cardiomyopathy* **High risk**: Severe valvular disease, prior MI, PCI or CABG, age > = 80, prior anthracyclines or radiation, stable angina, LVEF < 50%, or ≥ 5 points of medium risk factors **Moderate risk**: Medium risk factors with a total of 2–4 points **Low risk**: No risk factor or 1 medium risk factor **Medium risk factors**: borderline LVEF 50–54% (2 points), age 65–79 (2 points), HTN (1 point), DM (1 point), smoker (1 point), obese (1 point), elevated cTn (1 point), elevated NP (1 point)	**Very high**: very high baseline risk, doxorubicin ≥ 400 mg/m^2^, RT > 25 Gy MHD, RT > 15–25 Gy MHD + doxorubicin ≥ 100 mg/m^2^ **Early high** (< 5 years after therapy): high baseline risk, moderate to severe CTRC, doxorubicin 250–399 mg/m^2^, high-risk HSCT **Late high**: RT > 15–25 Gy MHD, RT 5–15 Gy MHD + doxorubicin ≥ 100 mg/m^2^, poorly controlled CV risk factors **Moderate**: moderate baseline risk, doxorubicin 100–249 mg/m^2^, RT 5–15 Gy MHD, RT < 5 Gy MHD + doxorubicin ≥ 100 mg/m^2^ **Low**: low baseline risk and normal end of therapy cardiac assessment, mild CTRC with recovery, RT < 5 Gy MHD, doxorubicin < 100 mg/m^2^

The HFA developed a risk scoring system specific to each cardiotoxic cancer therapy utilized during baseline cardiovascular risk assessment [62•]. Patients receive both qualitative (medium, high, very high) and quantitative scores that may guide biomarker surveillance. Examples in table provided for treatment with anthracyclines. The ESC 2022 guidelines adopted this risk tool for baseline risk assessment, while specifying additional risk factors during and after cancer therapy [6••]

*CABG* coronary artery bypass grafting, *cTn* troponin, *ESC* European Society of Cardiology, *Gy* gray, *HF* heart failure, *HFA* Heart Failure Association of the ESC, *ICOS* International Cardio-Oncology Society, *MHD* mean heart dose, *MI* myocardial infarction, *NP* natriuretic peptide, *PCI* percutaneous coronary intervention, *RT* radiation therapy, *TKI* tyrosine kinase inhibitor

^*^Other very-high risk factors for other cancer therapies include anti-HER2 therapy (baseline HF or cardiomyopathy, prior trastuzumab cardiotoxicity), vascular endothelial growth factor (baseline HF or cardiomyopathy, arterial vascular disease), proteasome inhibitors (baseline HF or cardiomyopathy, prior proteasome inhibitor cardiotoxicity, venous thrombosis, cardiac amyloidosis, arterial vascular disease), and multi-targeted kinase inhibitors (arterial vascular disease, arterial thrombosis with TKI)

### Emerging Biomarkers

GDF-15, MPO, gal-3, and other novel biomarkers have also been investigated in those receiving anthracycline therapy. In one cohort of patients treated with anthracyclines and trastuzumab, there was an early association between cardiotoxicity, troponin, and MPO [63]. At 15-month follow-up, MPO remained an important predictor of cardiotoxicity, while GDF and PlGF were also associated with cardiotoxicity at this later time point [64]. It is unknown whether these findings can be generalized to anthracycline treatment alone or only to those receiving both anthracyclines and trastuzumab. However, a recent meta-analysis did show that doubling of GDF-15 and gal-3 were associated with an increased risk of anthracycline-induced cardiotoxicity at 3 months (ejection fraction < 50–55% or 10%-point decrease) [65].

Early studies have additionally identified a few microRNA candidates as potential cardiotoxicity biomarkers. MicroRNA146a, MiRNA 140-5p, and MiRNA-377 have all been linked to cardiomyocyte death and/or mortality in animal models of doxorubicin cardiotoxicity [66–68], although more conclusive studies are needed regarding pathophysiology, measurement variability, and validation prior to clinical use.

## HER2 Directed Therapies

Human epidermal growth factor receptor 2 (HER2) is overexpressed in certain solid tumors, especially breast cancer, promoting proliferation, growth, and survival. HER2 inhibitors (trastuzumab, pertuzumab, lapatinib, neratinib) are the cornerstone for treatment. The cardiotoxic effects of HER2 antagonists are likely due to interruption of HER2 signaling on the cardiomyocyte, whose normal function appears to be integral to healthy cardiac adaptation [69]. LV dysfunction is not dose-dependent and is usually reversible after withdrawal of medication or starting guideline-directed medical therapy for LV dysfunction or HF [70–72]. Recent studies have actually shown that patients with mild LV dysfunction (LVEF > 40%) can often be continued safely on treatment with HER2 antagonists [73]. Incidence of HF with trastuzumab monotherapy ranges from 1 to 4% in clinical trials with an incidence of LV systolic dysfunction of 10–15% [5].

Biomarkers in trastuzumab cardiotoxicity have yielded mixed results partly because of concurrent or prior anthracycline therapy, different assays and thresholds, and inconsistent follow-up strategies. The biomarker substudy of the HERA trial measured both troponin and NT-proBNP in 533 women with HER2 + breast cancer (BC) receiving trastuzumab [8]. Though NT-proBNP has higher sensitivity than troponin in detecting new LVD following trastuzumab treatment, neither biomarker reached statistical significance [8]. Cardinale et al. showed that TnI elevation can be an important marker in identifying those who will develop HER-2 therapy cardiotoxicity and who are less likely to recover LVEF with HF treatment [74]. Importantly, most initial TnI elevations occurred after cycle 1 of treatment [74]. An observational study of 66 patients receiving trastuzumab found that NT-proBNP was significant associated with developing cardiotoxicity [75]. Other studies suggest hs-CRP may predict HER2 cardiotoxicity [76].

Given the lack of consistent data, there are no specific guidelines for cardiac monitoring during HER2-therapy in asymptomatic patients. The 2022 ESC guidelines recommend baseline measurement of NP and troponin in those receiving HER2 therapy to help identify those at increased risk during treatment, with consideration for monitoring while on therapy (Table 1) [6••]. Biomarkers continue to be investigated as a potential adjunct to regular imaging and may play an important role in patients with reduced access to imaging such as in the novel coronavirus-19 pandemic [77].

## Immune Checkpoint Inhibitors

ICI (e.g., pembrolizumab, ipilimumab, nivolumab) are a quickly growing class of immunomodulatory treatments that promote T-cell mediated destruction of tumor cells by inhibiting various binding sites (e.g., PD-1, PD-L1, and CTLA-4). ICI have been linked to accelerated atherosclerosis and associated CV events [78], but the most concerning cardiotoxicity is myocarditis, an uncommon but potentially fatal complication associated with activation of the immune system. Since myocarditis can respond to treatment, especially if found early, there is interest in developing screening algorithms incorporating troponin levels. In a multi-center registry of patients with ICI-associated myocarditis, 94% of patients had elevated troponin levels, although selection bias is a concern in registry-based research [79]. An elevation of troponin greater than the 99th percentile of the upper limit of normal is included as part of the minor criteria for the diagnosis of ICI-associated myocarditis [80].

The 2022 ESC guidelines recommend baseline troponin and NP assessment prior to ICI treatment and consideration for troponin measurements before doses 2, 3, and 4 and then every 3 doses, thereafter (Table 1) [6••]. While many organizations agree on baseline assessment of cardiac biomarkers for reference during treatment and risk assessment, the utility of troponin surveillance in asymptomatic patients during ICI treatment is less clear, and no consensus has been reached [81]. Troponin cannot differentiate ICI-induced myocarditis from other much more common causes of myocardial injury. Its measurement can potentially facilitate early diagnosis and prompt early intervention of serious adverse cardiac events including ICI-associated myocarditis. Importantly, there is no evidence that cancer therapy decisions should be based on a troponin elevation alone without any other evidence of clinically significant disease, especially given the tremendous life-prolonging impact that immunotherapy can exert on several cancer types.

## Multiple Myeloma and Proteasome Inhibitors

A foundational treatment of multiple myeloma, proteasome inhibitors (PI) lead to apoptosis by preventing degradation of intracellular proteins. PIs such as Ixazomib and bortezomib act as reversible inhibitors while carfilzomib is an irreversible selective inhibitor [82]. PIs have been associated with LVSD/HF in the first 3 months of therapy, particularly carfilzomib [7]. In a cohort of 95 patients with relapsed multiple myeloma receiving bortezomib or carfilzomib, elevation in NPs were common and predicted subsequent cardiovascular adverse events (symptomatic HF, acute coronary syndrome, symptomatic arrhythmia, venous thromboembolic events, pulmonary hypertension, grade 3 hypertension, or cardiac chest pain requiring treatment) [7]. Subsequently, the 2020 HFA and ESC position statement recommends considering NP measurement at baseline and during the first few cycles, particularly if the patient is being treated with carfilzomib [5].

## Cardiac Amyloidosis

Related to multiple myeloma, AL amyloid has a propensity for cardiac involvement, and baseline troponin and NP assessment have long been cornerstones for staging and prognosis [83]. More recently, it has also been noted that a 30% reduction in NP with AL treatment is associated with better survival and clinical outcomes, though the prognostic value of NT-proBNP is less certain in those with an eGFR < 15 mL/min/1.7m^2^ [9, 84–86]. Thus, NP can also be used to help track response to treatment. Current criteria in assessing AL amyloidosis response to therapy are based on light chain, creatinine, and NT-proBNP level, and current guidelines recommend biomarker assessment every 3–6 months [87, 88].

## Carcinoid Heart Disease

Carcinoid syndrome is a rare endocrine neoplasm that arises mostly from enterochromaffin cells and has it effects mediated by vasoactive substances like serotonin [89]. Patients with carcinoid syndrome can have tricuspid and pulmonic valve involvement (20–50%) with rare (< 10%) left-sided valve involvement as the vasoactive substances are deactivated in the lungs [90]. Among patients with carcinoid syndrome, a NT-proBNP level cutoff of 250 pg/mL had a 92% sensitivity and 91% specificity for carcinoid heart disease (CHD) [91]. Aside from predicting CHD, NT-proBNP also correlates well with survival [92]. Chromogranin A (CgA) has been found to be sensitive for CHD, but with low specificity [92]. Given current evidence, NT-proBNP level and echocardiography are recommended to screen for CHD in patients with carcinoid syndrome even in the absence of symptoms or signs of valvular heart disease [93, 94].

## Radiation-Induced Cardiovascular Disease

Over half of patients with thoracic cancer will be treated with radiation therapy (RT), which can result in incidental radiation to cardiac tissue. Consequently, an estimated one-third of thoracic cancer patients undergoing RT will develop radiation-induced cardiovascular disease (RICVD), which can include coronary artery disease, conduction abnormalities, myocardial fibrosis, peripheral vascular disease, and pericarditis [95]. The underlying pathophysiology of RICVD involves both direct damage to nucleic acids and biomolecules and indirect cellular injury via generation of reactive oxygen species, danger-associated molecular patterns, and other inflammatory factors [96]. While cardiomyocytes are relatively radioresistant, coronary endothelial cells are particularly sensitive to radiation injury, and therefore, coronary endothelial dysfunction is believed to be a critical initial step in RICVD [97]. Despite early radiation-induced cardiac changes, overt RICVD may not be diagnosed in patients until years after RT. Therefore, identification of RICVD biomarkers is a critical step in improving early detection, surveillance, and treatment of cardiac dysfunction in thoracic cancer patients treated with RT.

Several conventional markers of cardiac damage have been investigated in the context of RICVD. In female patients with left-sided breast cancer, troponin can be elevated within circulation immediately and 3 months post-RT [71, 98, 99], with troponin-I levels at 3 months post-RT correlating with mean radiation heart dose [98]. Similarly, BNP and NT-proBNP also can increase within 1 year of RT in breast cancer patients and vary with cardiac radiation dose [100, 101]. However, troponin and NPs elevations post-RT have not been consistently correlated with cardiac outcomes to date.

Beyond these conventional cardiac markers, there are several emerging biomarkers of RICVD under investigation. Emerging “omics” (e.g., genomics, metabolomics) tools hold potential for identifying new RICVD biomarkers, as alterations in nucleic acids and cellular metabolism are among the first to occur with radiation deposition [102, 103]. Other emerging biomarkers being applied to RICVD surveillance include lipopolysaccharide binding protein, which has been found to positively correlate with cardiac dose and post-RT diastolic function, gal-3, and pro-inflammatory cytokines (TNF-a, IL-1, IL-6) [71, 103–105]. Increased ST2 levels following adjuvant radiotherapy in chemo-naïve breast cancer patients have also correlated to worsening GLS; however, clinical outcomes are unknown [106]. Combining multiple plasma markers, or alternatively, a plasma biomarker with echocardiographic parameters such as global longitudinal strain or LV function, may improve RICVD prediction [63, 107]. There are no specific recommended screening guidelines for using biomarkers to detect RICVD at this time.

## VEGF Inhibitors

Vascular endothelial growth factor (VEGF) inhibitors prevent activation of cellular processes promoting angiogenesis and are commonly used in the treatment for renal cell carcinoma as well as metastatic colon and gastric cancer [108]. Hypertension is a common CV side effect of VEGF inhibitors. The resultant increase in afterload and simultaneous use of other cardiotoxic anti-cancer therapies puts patients at higher risk of LV dysfunction [5, 109]. In a study of 159 patients with renal cell carcinoma treated with VEGF inhibitors, NP were associated with LV dysfunction [110]. There is currently no data to support troponin monitoring in patients treated with VEGF inhibitors as it was unable to predict LVSD, HF, or ischemic events in one small study [110]. NP on the other hand may precede LVEF reduction or clinically relevant HF in cancer patients treated with anti-VEGF therapy [5, 110]. Current expert recommendations include blood pressure monitoring at every clinic visit with NP assessment at baseline, 1 month following treatment, and every 3 months thereafter [5]. The 2022 ESC guidelines recommend NP and troponin measurement at baseline and every 4 months in the first year of treatment in moderate-risk patients, and at baseline, 4 weeks, and every 3 months in the first year of treatment in high and very-high risk patients (Tables 1 and 2).

## Conclusions

Cardiac biomarkers, primarily troponin and NP, have become integral in assessing baseline risk and monitoring for subclinical cardiotoxicity in patients during and after treatment with high-risk cancer therapy. Based on available evidence, biomarkers are strongly featured in recommendations for management of patients on anthracyclines, anti-HER2 antagonists, immunotherapy, proteasome inhibitors, and VEGF inhibitors as well as patients being evaluated for amyloidosis. Nevertheless, many questions remain regarding optimal protocols and prognostic algorithms in various types of cancer and cancer therapeutics. Numerous novel biomarkers are also emerging with promise in the world of cardio-oncology such as MPO and micro-RNAs. Further research is needed to better elucidate their clinical utility to predict cardiovascular events and refine prognosis in patients with cancer or a history of cancer.
